# Are there cognitive and neuroimaging signatures in long COVID?

**DOI:** 10.1093/braincomms/fcad189

**Published:** 2023-06-28

**Authors:** Jordi A Matias-Guiu, María Díez-Cirarda

**Affiliations:** Department of Neurology, Hospital Clínico San Carlos, Health Research Institute ‘San Carlos’ (IdISCC), Universidad Complutense de Madrid, Madrid, Spain; Department of Neurology, Hospital Clínico San Carlos, Health Research Institute ‘San Carlos’ (IdISCC), Universidad Complutense de Madrid, Madrid, Spain

## Abstract

This scientific commentary refers to ‘Markers of limbic system damage following SARS-CoV-2 infection’, by Thomasson *et al.* (https://doi.org/10.1093/braincomms/fcad177)

**This scientific commentary refers to ‘Markers of limbic system damage following SARS-CoV-2 infection’, by Thomasson *et al.* (https://doi.org/10.1093/braincomms/fcad177)**

Infection by SARS-CoV-2 is associated with several long-term consequences. Among these, cognitive disturbances and fatigue are two of the most salient and disabling symptoms according to the different definitions of post-COVID syndrome, post-COVID condition or long COVID.^1^ In the last few years, several studies have characterized the cognitive profile of patients with post-COVID syndrome. Specifically, the involvement of attention and processing speed is generally the most impaired cognitive domain, mainly followed by deficits in episodic memory and executive functions.^2,3^ This clinical syndrome affects women in middle age more frequently, and it is not limited to patients with severe acute illness.

Subsequently, other studies have detected several neuroimaging changes in patients after COVID-19. In a longitudinal study including participants from the UK Biobank, patients infected by SARS-CoV-2 showed a greater reduction in grey matter thickness in the orbitofrontal cortex and parahippocampal gyrus.^4^ In a cross-sectional study including patients with cognitive symptoms after COVID-19 evaluated at 1 year, reduced volumes in these regions were also detected. Furthermore, patients with COVID-19 showed reduced connectivity between the orbitofrontal cortex and cerebellum and between the left and right parahippocampal regions compared with controls. In addition, there were widespread changes in diffusion tensor imaging. Importantly, grey matter and functional connectivity changes were correlated with cognitive performance, especially in those tests most commonly impaired in these patients.^5^ Other studies have also confirmed widespread white matter diffusivity changes and predominant involvement of several regions, including the orbitofrontal cortex, hippocampi and parahippocampal regions, cingulate cortex and insula.^6^

Overall, previous studies provide evidence for the existence of cognitive dysfunction in patients with post-COVID syndrome. The cognitive profile shows a predominant involvement of attention deficits and processing speed,^7^ and neuroimaging studies using advanced techniques reveal the involvement of several regions and brain circuits, which, at the same time, have been linked with cognitive deficits. This growing knowledge suggests the need to characterize the cognitive consequences of COVID-19 further. The involvement of regions belonging to the limbic system supports the study of specific cognitive abilities linked to this system. In this regard, the recent article in *Brain Communications* by Thomasson *et al*.,^8^ examined the emotion recognition abilities in 105 patients 6–9 months after the acute SARS-CoV-2 infection. Alterations in recognition of emotion expressions, mostly fear, disgust and irritation, were detected in moderate and severe patients. These emotion recognition alterations were associated with decreased episodic memory and olfaction. In addition, the authors examined the functional brain connectivity correlates in a subgroup of 45 patients. Significant correlations between emotion recognition performance and cortico-subcortical-cerebellar networks, including areas of the limbic system, were observed. These findings provide novel evidence about the cognitive characteristics associated with long-term consequences of SARS-CoV-2 infection and confirm the involvement of the limbic system and associated brain regions and networks. Interestingly, the authors found different correlations (positive or negative) in functional connectivity according to the severity of the acute infection, suggesting different mechanisms of damage or compensation according to the initial severity.

A critical challenge in the research of patients with persistent symptoms after COVID-19 is the potential participation of several pathophysiological mechanisms. Mechanisms of neuroinflammation, autoimmunity, neurovascular dysfunction, viral neuroinvasion, failure of hippocampal neurogenesis, hypoxia during the acute phase and even reactivation of latent herpes virus have been hypothesized and at least partially supported.^9^ These mechanisms are not exclusionary, and a mechanism could predominate in some patients, while other patients may have several mechanisms implicated at the same time. In addition, cognitive symptoms are not necessarily associated with objective cognitive deficits in neuropsychological assessment because other factors (fatigue, neuropsychiatric symptoms) may also have a role. The heterogeneity of mechanisms and causes could also explain the existence of subgroups of patients with specific cognitive or neuroimaging changes.

The current evidence corroborates the existence of cognitive deficits in patients with post-COVID-19 persistent symptoms and their association with neuroimaging changes. Despite the heterogeneity of post-COVID syndrome and the difficulties specified above, cognitive and neuroimaging changes show a profile that has been replicated across different studies. A thorough characterization of cognitive dysfunction and its neuroimaging correlates is necessary to guide the diagnosis of post-COVID condition, the differential diagnosis with other disorders (including neurodegenerative diseases) and treatment. These findings warrant the investigation of the cognitive and neuroimaging longitudinal course, which is still largely unknown. Identifying cognitive and/or neuroimaging signatures of long COVID may have diagnostic relevance in clinical practice.
